# NT-proBNP detection with a one-step magnetic lateral flow channel assay

**DOI:** 10.1007/s00216-024-05223-x

**Published:** 2024-03-08

**Authors:** Dan Strohmaier-Nguyen, Carina Horn, Antje J. Baeumner

**Affiliations:** 1 Institute of Analytical Chemistry, Chemo- and Biosensors, University of Regensburg, 93053 Regensburg, Germany; 2 Roche Diagnostics GmbH, 68305 Mannheim, Germany

**Keywords:** Magnetic bead, Lateral flow assay, Immunoassay, Cardiac, One-step, Point-of-care

## Abstract

Point-of-care sensors targeting blood marker analysis must be designed to function with very small volumes since acquiring a blood sample through a simple, mostly pain-free finger prick dramatically limits the sample size and comforts the patient. Therefore, we explored the potential of converting a conventional lateral flow assay (LFA) for a significant biomarker into a self-contained and compact polymer channel-based LFA to minimize the sample volume while maintaining the analytical merits. Our primary objective was to eliminate the use of sample-absorbing fleece and membrane materials commonly present in LFAs. Simultaneously, we concentrated on developing a ready-to-deploy one-step LFA format, characterized by dried reagents, facilitating automation and precise sample transport through a pump control system. We targeted the detection of the heart failure biomarker NT-proBNP in only 15 µL human whole blood and therefore implemented strategies that ensure highly sensitive detection. The biosensor combines streptavidin-functionalized magnetic beads (MNPs) as a 3D detection zone and fluorescence nanoparticles as signal labels in a sandwich-based immunoassay. Compared to the currently commercialized LFA, our biosensor demonstrates comparable analytical performance with only a tenth of the sample volume. With a detection limit of 43.1 pg∙mL^−1^ and a mean error of 18% (*n* ≥ 3), the biosensor offers high sensitivity and accuracy. The integration of all-dried long-term stable reagents further enhances the convenience and stability of the biosensor. This lateral flow channel platform represents a promising advancement in point-of-care diagnostics for heart failure biomarkers, offering a user-friendly and sensitive platform for rapid and reliable testing with low finger-prick blood sample volumes.

**Graphical abstract:**

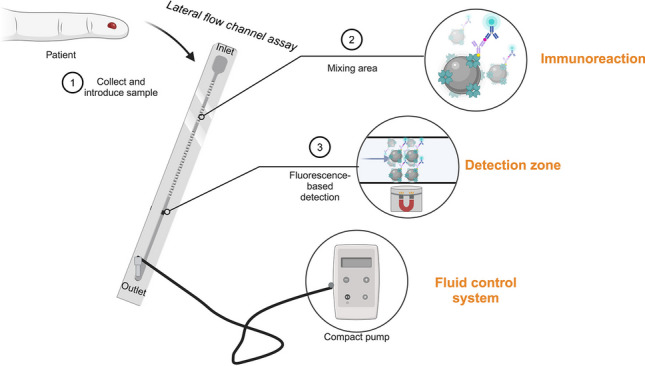

**Supplementary Information:**

The online version contains supplementary material available at 10.1007/s00216-024-05223-x.

## Introduction

According to the World Health Organization (WHO), cardiovascular diseases (CVDs) remain the most frequent cause of death globally [1]. Among all cardiovascular disorders of the heart and blood vessels, heart failure (HF) leads to the highest rate of mortality, morbidity, and health care costs [2]. HF represents the condition when the heart insufficiently provides blood and oxygen to the body organs and thus cannot meet the demands of the organs [3]. It describes a progressive condition and is grouped into four stages based on the magnitude and progression of the disease. HF starts with cardiovascular risk factors for left ventricular systolic followed by structural and functional changes of the cardiac system such as left ventricular hypertrophy and eventually leads to observable HF, dysfunction, and death [4]. Therefore, detection and intervention in early phases can prevent or decelerate disease progression and is of primary importance for the patient.

N-terminal prohormone of BNP (NT-proBNP) is one of the major biomarkers in HF due to its significance in diagnosis and treatment [5–7]. Abnormal levels of NT-proBNP are released in response to myocyte enlargement following left ventricle hypertrophy [8]. Early detection and monitoring of NT-proBNP level can diagnose and prevent the risk of LVH, LVSD, and HF and, thus, can protect the cardiovascular function and clarify the treatment strategy for the physician [9, 10]. The age-independent NT-proBNP value of 300 pg∙mL^−1^ is recommended as cut-off value for HF diagnosis, which had a sensitivity of 99% and a specificity of 68% [11]. Fast and reliable diagnosis of HF has been accomplished by commercially viable lateral flow assays (LFAs) in point-of-care testing (POCT). For example, the cobas h 232 NT-proBNP from Roche Diagnostics GmbH provides a sensitive platform that detects NT-proBNP in 150 µL whole blood with a detection limit of 60 pg∙mL^−1^ and a detection range of 60–9000 pg∙mL^−1^ [12]. In general, the LFAs consist of fleece fiber and membrane components, often employed as the drying substrate for probe-specific antibodies and the sample transport [13]. Both come with an innate high analyte and sample adsorption capacity, resulting in a notable requirement for sample volume, a relatively substantial dead volume, and an impairment of the sensor performance [14]. Therefore, the requirement of relatively large blood sample volume, attributed to sample-adsorbing materials like fleece and membrane, compromises the test’s performance and patient convenience. In addition, it necessitates the presence of skilled personnel to obtain blood from veins in a clinical setting, increasing the risk of complications and infection and reducing patient comfort and safety. Hence, the field of POCT is characterized by intense competition, driving a continuous demand for the rapid, cost-effective development of precise tests with low sample volumes. Developing POCT into minimally invasive finger-prick blood tests has presented challenges due to technological limitations. The primary hurdle in working with finger-prick volumes is that the target analyte for detection exists in lower concentrations compared to venous blood samples, which poses a difficulty in accurately detecting such minimal analyte levels. Predominantly, the challenge of maintaining assay performance has impeded the widespread commercial operation. However, this aspiration must remain a priority driven by the potential to offer techniques enabling ultra-sensitive detection with just finger-prick volumes that reduce the invasiveness and simplify the sample processing. As a result, this comforts the patient and enables diagnostics through lay personnel, thereby expanding medical care to rural areas and homecare.

Microfluidic platforms, in POCT, have gained a lot of attention due to their benefits, including the ability to work with small volumes of samples and reagents, seamless integration of multiple components, and fast reaction kinetics [15–17]. In addition, it is commonly acknowledged that microfluidic channels offer superior surface area to volume ratios [18, 19]. However, it should be noted that the detection area is often restricted to the miniaturized geometric structure of the channel, where the antibodies or other recognition molecules are fixed. Thus, due to the slow diffusion in the laminar flow, only a limited part of the sample can be captured, reducing the sensor’s performance [20]. To enhance the surface area and reduce the diffusion distance for binding, particles labeled with recognition elements can be introduced into the channels. This significantly reduces the diffusion distance and increases the surface area available for binding relative to the sample volume in the detection zone, increasing the assay signal [20, 21]. Especially, the inclusion of magnetic nanoparticles in LFAs and microfluidic systems plays a crucial role in performing biochemical assays for healthcare and diagnosis [20, 22–25]. MNPs contribute to the enhancement of various biosensing applications due to their distinctive superparamagnetic properties since they can function as both mobile substrates and detection markers simultaneously [26, 27]*.* The simple bio-functionalization with a variety of molecules makes them an ideal option for capturing and signaling label in immunoassays [28–30]*.* Here, we developed a novel LFA concept using an external suction pump for sample transportation and streptavidin-functionalized magnetic particles (MNPs) for the POC detection and quantification of NT-proBNP in whole blood. Since the assay requires only 15 µL whole blood and the pump can be included in a simple, portable support device, this new concept offers an alternative to membrane-based LFAs as it requires less sample volume and can thus easily be used for finger-prick analyses.

### Theory and operating principle

When particles move within a Newtonian flow of a microchannel, they are subjected to forces such as inertia lift and hydrodynamic drag [31]. The inertial lift force can be calculated by [32]

1 $${F}_{l}=\frac{{\rho }_{f}{U}_{m}^{2}{a}^{4}}{{D}_{h}^{2}}{f}_{L}\left(R{e}_{c}, {x}_{c}\right)$$

while *ρ*_*f*_, *μ*_*f*_, and *U*_*m*_ represent the density, dynamic viscosity, and mean flow velocity of the fluid transporting the particles, respectively. The diameter of the spherical microparticle is denoted by *a*, and *D*_*h*_ refers to the hydraulic diameter of the flow channel, which can be expressed as ${D}_{h}= \frac{2wh}{\left(w+h\right)}$ for a rectangular channel, where *h* is the height and *w* is the width of the channel. *f*_*L*_ represents the lift coefficient and is a function of the Reynolds number *Re*_*c*_ and the normalized cross-sectional position of the particle within the channel *x*_*c*_. The hydrodynamic drag force can be expressed by the following [33]:

2 $${F}_{d} = 3\pi {\mu }_{f} a\left({v}_{f} - {v}_{p}\right){f}_{D}\sim 3\pi {\mu }_{f} a({v}_{f} - {v}_{p})$$

In this context, the velocities of the fluid and microparticles are represented by *v*_*f*_ and *v*_*p*_, respectively. The coefficient for hydrodynamic drag force, denoted as *f*_*D*_, is dependent on the hydraulic diameter of the channel and the diameter of the particles, as well as the distance between the particle and the closest channel wall. For low concentrations of ferrofluid, such as those studied in this study, *f*_*D*_ can be considered equal to 1, since the magneto-viscous effect can be disregarded [34].

The MNPs were captured from the sample by applying an external magnetic field, using a circular permanent magnet. In this case, the particles are exposed to the magnetophoresis force [35, 36]:

3 $${F}_{m}= \frac{\Delta \lambda *{V}_{p}}{{\mu }_{0}}*\left(\nabla B\right)*B$$

In the given equation, *B* represents the magnetic flux density, *∇B* denotes the gradient of the externally applied magnetic field, *μ*_*0*_ refers to free space permeability, and *Δλ* represents the difference in susceptibility between the particle and the fluid*.* In general, the deflection process of the MNPs in the microchannel is the total of three forces: the magnetically induced force on the particle, *F*_*m*_, the hydrodynamic drag force *F*_*d*_, and the lift force *F*_*l*_ [37]:

4 $${F}_{defl}={F}_{m}+{F}_{d}+{F}_{l}$$

**Fig. 1:**
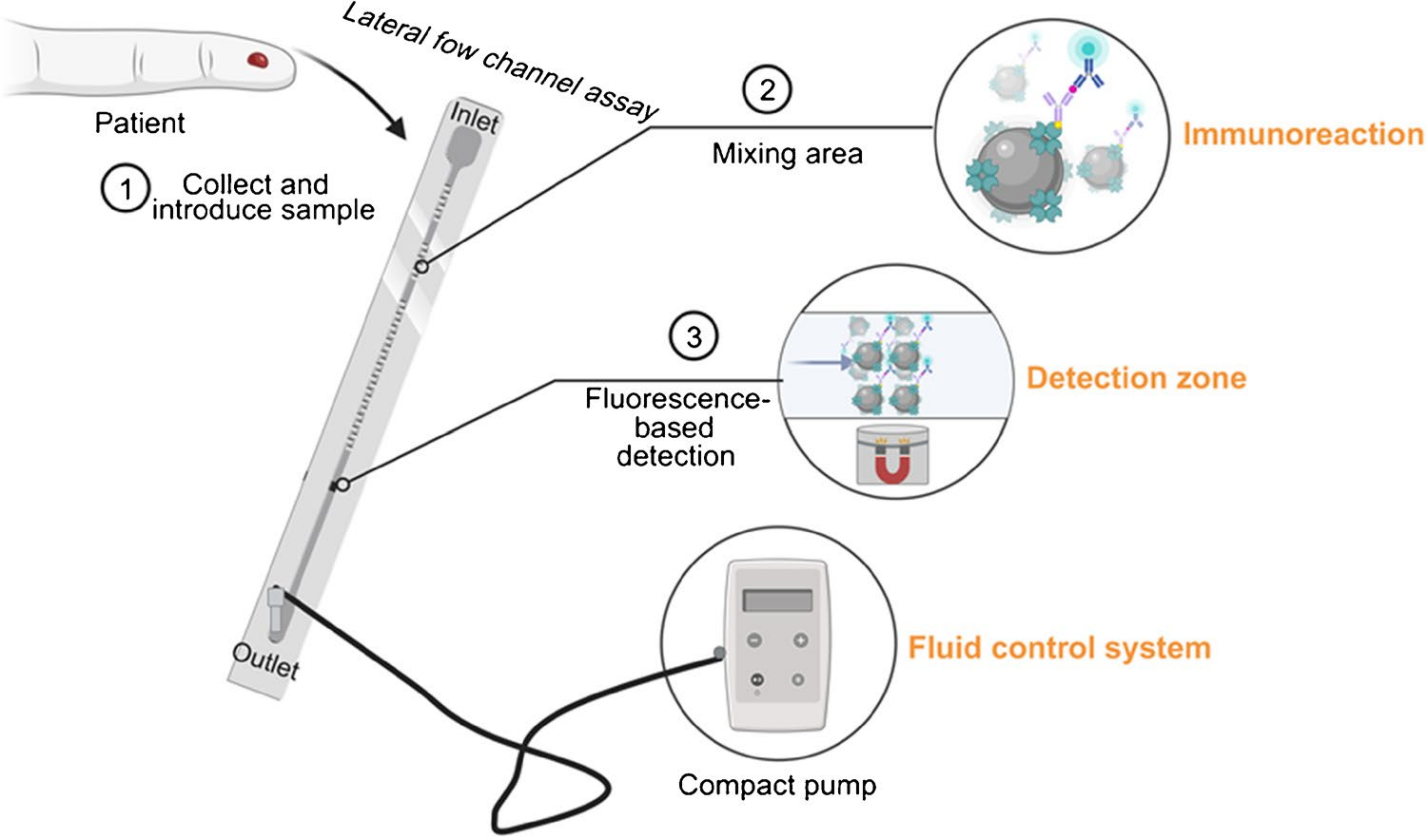
Schematic (not-to-scale) illustration of the sandwich-based fluorescence assay in a lateral flow channel with MNPs as capturing particles, an external magnet for fixing the MNPs in the detection zone, fluorescence nanoparticles as label, and a fluid control system for sample transportation. Upon sample application (15 µL) at the inlet (1), the dried reagents undergo rehydration and subsequent transport through the mixing area, initiating the immunoreaction process (2). Positioned within the detection zone, an external magnet captures the MNP-immunosandwich complex (3), enabling the quantification of analyte-dependent fluorescence. Adapted from “Microfluidic Device,” by BioRender.com (2023). Retrieved from https://app.biorender.com/biorender-templates

In this study, streptavidin magnetic nanoparticles were utilized as mobile capturing particles for the detection of NT-proBNP in a sandwich-based fluorescence lateral flow channel assay (Fig. 1). The MNPs were able to capture the analyte in a sandwich complex in undiluted blood samples through streptavidin–biotin interaction. The immunoassay utilizes a magnetic separation where the MNPs are pulled from the sample stream to the detection zone by a stationary external magnet, whereas the unbound sample solution flows further into the waste outlet. Subsequently, the sandwich complex captured in the detection zone was quantitatively analyzed using fluorescence microscopy.

## Materials and methods

For the NT-proBNP immunoassay, all reagents unless stated otherwise were provided by Roche Diagnostics GmbH (Mannheim, Germany) and Sigma-Aldrich. Mouse anti-human NT-proBNP antibodies were used as biotinylated capture antibody (capture Ab), and the detection antibody for the formation of the sandwich complex was identical to the antibodies in the fifth-generation hs-BNP assay in the Elecsys®/cobas e™ platform. NT-proBNP antibody-modified fluorescence nanoparticles (Ab-fluorescent NPs) were used for fluorescence signal generation. For this reason, latex particles (200 nm, Thermo Fisher Scientific) were dyed with squaraine (λ_ex_ = 652 nm, λ_em_ = 665 nm) and coupled with the detection antibody via EDC and sulfo-NHS chemistry. For capturing the biotinylated sandwich complex, streptavidin-conjugated magnetic particles (MNPs) (diameter (d) = 0.3 µm, 3 µm; 10% (w/v)) in combination of a round external magnet (*r* = 6 mm) were used.

HEPES-buffered saline (HBS) consisted of 50 mM HEPES, 150 mM NaCl, 3 mM EDTA, and 0.05% (w/v) Tween 20 and was adjusted to pH 7.4. The dispensing buffer was prepared with 50 mM HEPES, 1% (w/v) BSA, 8% (w/v) sucrose, 0.15% (w/v) Synperonic PE/P84, and 0.024% (w/v) sodium azide and was adjusted to pH 7.4. The dextran dispensing buffer was prepared with 50 mM HEPES, 1% (w/v) BSA, 8% (w/v) dextran, 0.15% (w/v) Synperonic PE/P84, and 0.024% (w/v) sodium azide and was adjusted to pH 7.4. HEPES blocking buffer was prepared with 50 mM HEPES, 2.5% (w/v) BSA, and 0.15% (w/v) Synperonic PE/P84 and was adjusted to pH 7.4.

### Fabrication of lateral flow channel immunoassay and drying of reactive reagents

The LFA consists of a three-layer built-up: (1) the support foil (Melinex® 329 foil, 175 µm (Dupont Teijin Films, www.dupontteijinfilms.com)), (2) the spacer foil (Melinex®329 foil, 250 µm, with double-sided adhesive tape (Henkel Adhesives, www.henkel-adhesives.com) on both sides), and (3) the cover foil (Hostaphan RN 100, 125 µm (Mitsubishi Polyester Film, www.m-petfilm.de)) (see Supplementary Information (ESM) Fig. S1). The capillary (dimension, 90 mm × 1.5 mm × 0.28 mm) was cut in the spacer foil, and the inlet and outlet (*r* = 0.5 mm) were cut in the cover foil with the laser MicroLine 6000 P (www.lpkf.com) at a frequency of 30 kHz and 3W.

To prepare the ready-to-go, one-step immunoassay, the reactive reagent capture Ab and detection Ab-fluorescence NP were mixed with the dispensing buffer to give a 2.5 µg∙mL^−1^ and 2% (w/v) solution, respectively, and a sucrose concentration of 6% (w/v). For the MNPs, the dispensing buffer was adjusted through replacing sucrose by additives such as trehalose, dextran, Tween 20, Tween 80, and polyethylene glycol. The drying and the additives’ content were optimized with respect to prevention of aggregation and high assay signal. The MNPs were mixed with the adjusted dispensing buffer to give a 2% (w/v) MNP solution. Capture Abs, Ab-fluorescent NPs, and MNPs were applied separately (2 µL each) onto the support of the immunoassay and dried in a drying cabinet at 50 °C for 10 min (FED 400 E2, www.binder-world.com). The support, spacer, and cover foil were then bond together as depicted in the ESM (Fig. S1).

### Analysis of the capturing efficiency of the applied magnetic field

To investigate the capture efficiency of the small and large MNPs by the magnet, 200 µl of MNPs 2% (w/v) in HEPES buffer was transported in different flow rates to the magnet in the detection zone of the lateral flow channel. The magnet is placed at a distance of 175 µm from the bottom wall of the channel and at a distance of 6.5 cm from the application zone to mitigate the impact of initial forces that may arise upon applying the sample to the sensor. After capturing the MNPs, the downstream sample was collected, and its transmittance was analyzed in the photometer.

### Analysis of the biotin-binding capacity of the MNPs

A biotin-5-fluorescein working solution (1 µmol∙L^−1^) was prepared in HEPES buffer. Five hundred microliter of the biotin-5-fluorescein working solution was mixed with 50 µl of MNPs (0.3 µm, 2% (w/v)) and 50 µl of MNPs (3 µm, 2% (w/v)) and incubated in the dark at room temperature for 15 min. Afterwards, the MNPs were pelleted by centrifugation (5000 × g for 10 min) and the fluorescence of the supernatant was measured (λ_ex_ = 490 nm, λ_em_ = 524 nm). The biotin-binding capacity (pmol biotin/mg MNP) was then calculated from the difference of the fluorescence intensity in the presence and absence of the MNPs.

### Sample preparation

Human blood samples were drawn by venipuncture from volunteers and provided by Roche Diagnostics GmbH in compliance with safety and ethical regulations. The hematocrit was obtained by micro-hematocrit capillary tubes. The blood samples were collected in EDTA-K3 vacutainer tubes and were used within a day.

### NT-proBNP assay

A sandwich immunoassay with spiked samples was conducted to investigate the assay performance of the bioassay. For analysis, NT-proBNP biomarker samples were prepared in HBS buffer, human plasma, and human whole blood through dilution of a stock solution to produce concentrations of 7.5, 15, 30, 60, 125, 250, 500, 1000, 2000, 4000, 6000, and 9000 pg∙mL^−1^. A volume of 15 µL of the spiked sample was applied on the sample application area. The sample was transported with a flow rate of 2 µL/min by the external vent control. The immunoassay was performed at room temperature, and fluorescence signals could be obtained after 10 min. For the calculation of the limit of detection (LOD), the logistic fit parameter for the lower curve asymptote A and the standard deviation of the blank SD (blank) was used:

5 $$LOD = A + 3.3 * SD(blank)$$

The concentration of NT-proBNP in the sample is determined by correlating the fluorescence intensity to a calibration curve constructed using known concentration standards.

The functionality of the dried reagents was assessed by replicating the bioassay over a 8-week period. To conduct these measurements, the sucrose-based dispensing buffer was utilized to dry the capture Ab and Ab-fluorescent NPs, and the dextran-based dispensing buffer was utilized to dry the MNPs for 10 min at 50 °C. The one-step LFA was stored at 4 °C in an airtight capsule.

### Supporting instrumentation

For any of these applications, the microfluidic assay will require supporting instrumentation to control reagent and sample transport, along with fluorescence detection. The embodiment includes a customized fluid control equipment (suction pump system) that is controlled by a program code, provided by Roche Diagnostics GmbH (Mannheim, Germany). The fluid vent control was mounted on top of the outlet port. All fluorescence images were acquired using a fluorescence microscope, LINOS lens (www.excelitas.com), HTC camera with a Sony CCD sensor ICX285AL (www.sony.com), XENON XBO R 100W/45 OFR lamp (www.osram.com), 633-nm excitation filter and 685-nm detection filter (www.semrock.com), and an image processing software provided by Roche Diagnostics (Mannheim, Germany). The fluorescence images were taken with an exposure time of 25 ms. Data was analyzed with Origin 2021.

## Results and discussion

Finger-prick blood tests hold significant promise as POCTs due to their convenience in sample collection and the minimal sample volume needed. This not only enhances patient comfort but also eliminates the need for trained personnel for the venous blood withdrawal. The fleece fibers in conjugate pads, commonly used as a drying matrix for probe-specific antibodies in traditional LFAs, have an inherent high analyte and sample adsorptive capacity, which leads to a significant demand for sample volume, a relatively large dead volume, and a reduction of the sensor performance. The same principle applies to membrane materials, which enables the sample transport and the immobilization of capture ligands for proteins. Considering the commercial application of a finger-prick blood test without sample-adsorbing components, we studied the analyte capture efficiency and flow behavior of MNPs in a straight channel for a fluorescence-based immunoassay. The conjugate pad was replaced by dry-spotted Abs, whereas streptavidin magnetic nanoparticles and the pump system replaced the membrane’s function.

### Biotin-binding capacity

We examined the impact of different sizes and surface areas (sa) of MNPs on their ability to improve diffusion-limited interactions in polymer channel lateral flow assays. First, we compared the biotin-binding capacity for biotin-5-fluorescein between small MNPs (d = 0.3 µm, sa = 4.57 × 10^−6^ m^2^∙mg^−1^) and large MNPs (d = 3 µm, sa = 3.81 × 10^−8^ m^2^∙mg^−1^) (ESM, Fig. S2, dark blue). Interestingly, both MNPs provided similar biotin-binding capacity of 2600 µmol∙g MNP^−1^ and 2400 µmol∙g MNP^−1^, respectively. Despite smaller MNPs having a larger surface area, they may exhibit a lower streptavidin coverage density compared to their larger counterparts, suggesting that, in this case, the size of the MNPs does not affect the binding capacity. In addition, to account for the factor of steric hindrance of antibodies, the fluorescence signal of the immunoassay in the LFA was investigated (ESM, Fig. S2, light blue). Similar fluorescence signals were obtained for both MNPs for a given analyte concentration, using the same MNP concentration. These findings suggest that interestingly, the size of the particles and surface area do not significantly affect factors such as biotin-binding capacity and steric hindrance during the immunoreaction.

### Capture efficiency by external magnet

**Fig. 2:**
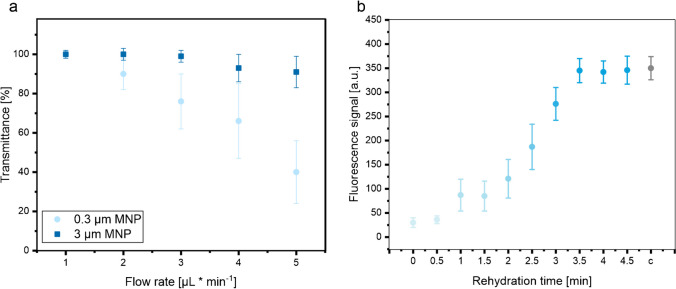
Optimization of the capture efficiency by the external magnetic field in the lateral flow channel in dependency of the particle size and flow rate. Plot of the transmittance of the sample against the flow rate and particle size (**a**). Two hundred microliter of MNP (2% (w/v)) in HEPES buffer was transported with different flow rates through the channel, and the transmittance of the sample was then analyzed in the photometer. Plot of the assay signal against the rehydration time of the dried reagents (**b**). Control c represents the assay signal with Ab-fluorescent NPs, capture Ab, and MNPs in solution. Standard deviations were calculated based on three parallel measurements on three different lateral flow channels (*n* = 3)

The primary goals of the magnetophoretic capture process are to decrease the count of untrapped particles and to ensure that the flow rate is adequate for achieving optimal capture efficiency and collection quality during magnetic separation. The capture efficiency of the MNPs was determined by measuring the transmittance of the supernatant after the capture process in the lateral flow channel, taking into account the particle size and flow rate (Fig. 2a). A 100% transmittance of the supernatant is defined as the state where all MNPs have been captured by the magnet, resulting in a maximum capture efficiency and a MNP-free supernatant. Conversely, if MNPs are not adequately captured by the magnet, the supernatant will contain MNPs, leading to a decrease in transmittance. As expected, for both MNPs, the capture efficiency decreased with increasing flow rate. Moreover, at the lowest flow rate, both MNP samples demonstrated full capturing. Interestingly, at higher flow rates, differences between nanoparticles and microparticles were observed. The transmittance of the small MNPs strongly decreased with increasing flow rate, while the transmittance of the large MNPs decreased only slightly. The optimal flow rate for the 0.3 µm particles (1 µl∙ml^−1^) was generally lower than that for the 3 µm magnetic particles (3 µl∙ml^−1^). The larger particles with greater susceptibility experienced a greater magnetic attraction and therefore endured higher flow velocities. This correlates to earlier findings indicating a dependency of particle size or magnetic core size of coated particles on flow behavior under flow conditions [35, 37]. As a result, the selection of larger MNPs for subsequent experiments was driven by the potential to apply a wider range of flow rates in the assay. Furthermore, we enhanced the slightly lower specific surface area of these larger MNPs and their increased steric hindrance by augmenting the particle count.

### Development of a dry-reagent lateral flow channel immunoassay

The biotinylated polyclonal capture antibodies and monoclonal probe antibodies employed in this study were identical to the antibodies of Roche’s fifth-generation hs-BNP assay in the Elecsys®/cobas e™ platform. Hence, the antibodies’ selectivity and specificity were presumed to be adequate without conducting additional tests. The initial experiments aimed to assess the viability of drying probe antibodies and probe antibodies labeled fluorescent NPs and MNPs. Since Lutz et al. demonstrated that the functionality of the antibodies could be effectively preserved after the drying and rehydration process [38], here only the effect of drying on MNPs was investigated. Nanoparticles are known to easily adsorb or aggregate irreversibly due to the reduced spacing and the compromised electrostatic repulsion between the nanoparticles during the drying process. This impairs the biological functionality of the particles [39–41]. Thus, to prevent aggregation and maintain the long-term functionality, various additives such as polyethylene glycol (PEG), surfactants such as Tween 20 and Tween 80, and various sugars were included in the dispensing matrix (ESM, Fig. S3). After drying 2 µL of MNPs (2% (w/v)) in the dispensing buffer on the PET support and rehydration with 15 µL of NT-proBNP-spiked HEPES (c_[analyte]_ = 1 ng∙mL^−1^), we observed the formation of aggregates and significant irreversible adsorption of MNPs onto the PET support with all additives except of dextran. Drying the MNPs in only HEPES buffer (blank) diminished the signal to only 5%. In all other drying buffers, the fluorescence signal dropped to only 55% of the original signal. The drying of MNPs in the dextran-based matrix resulted in the most favorable fluorescence signals, indicated by a signal drop to only 92% in comparison to the control. This observation suggests that dextran successfully prevented the irreversible adsorption of MNPs on the support and minimized aggregation, indicating its efficacy in maintaining the integrity of the MNPs during the drying process. Subsequently, the rehydration time of the dried reagents was assessed by exposing them to different durations of rehydration in the channel. Here, we determined the optimal condition with respect to the reagents effective functionality and fluorescence signal in the immunoassay (Fig. 2b). The most favorable signal was obtained by rehydrating the dried reagents with 15 µL NT-proBNP-spiked HEPES buffer (c_[analyte]_ = 1 ng∙mL^−1^) for 3 min. In fact, the fluorescence signal was minimally affected by drying when compared to the control, which represents Ab-fluorescent NPs, capture Abs, and MNPs in solution. This suggests that the rehydrated probe antibodies retain their biological function and that the Ab-fluorescent NPs do not form aggregates that would hinder their fluorescence quantification.

### Optimization of analyte–capture antibody interactions

**Fig. 3:**
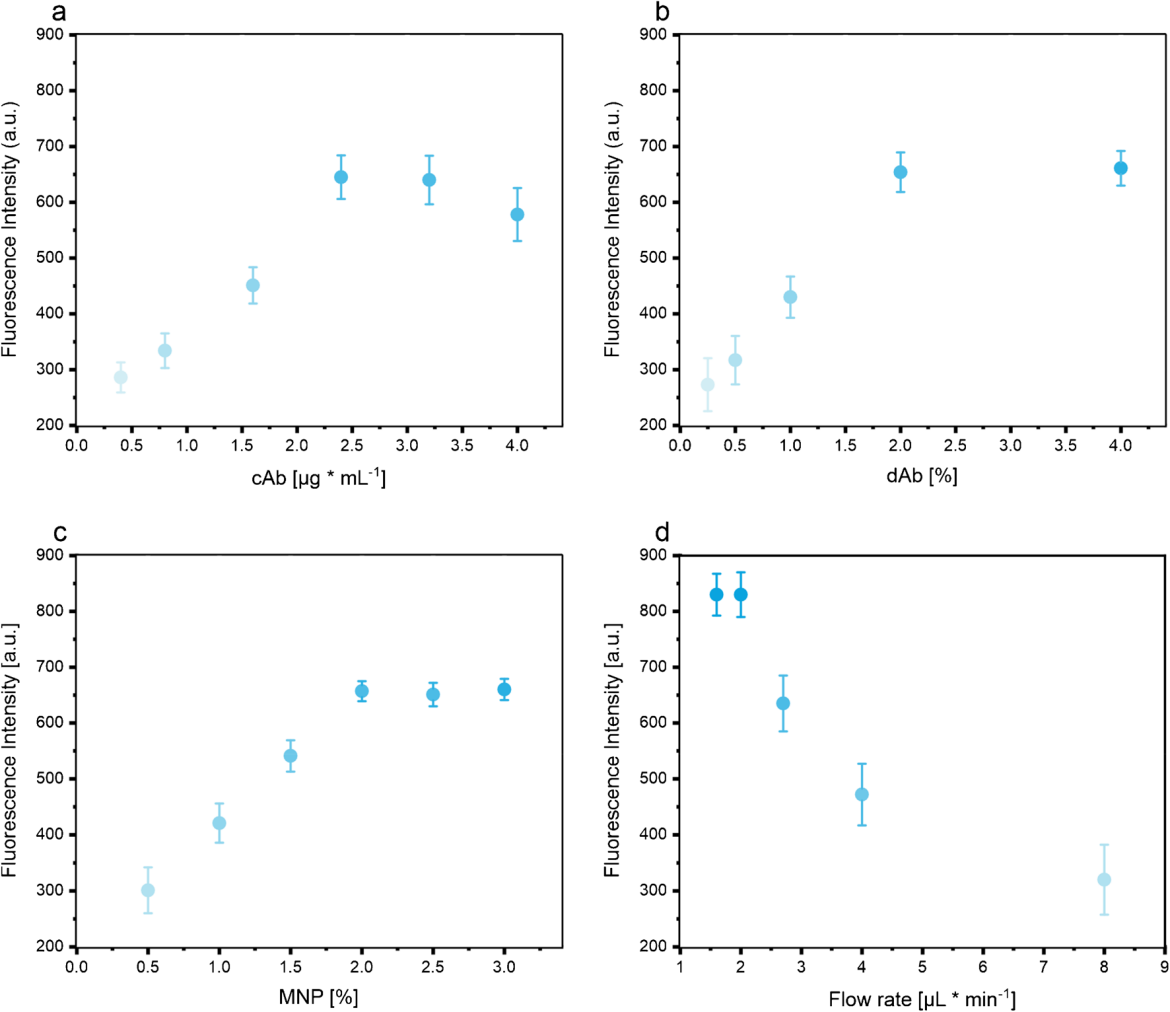
Optimization of the immunoassay by varying the concentration of the dried reagents and the flow rate. Plot of the assay signal against the capture antibody (cAb) concentration (**a**), plot of the assay signal against the Ab-fluorescent NP (dAb) concentration (**b**), plot of the assay signal against the MNP concentration (**c**), and plot of the assay signal against the flow rate (**d**). Error bars represent mean values ± 1 σ (n = 5)

It is known that heterogeneous immunoassays can have lengthy detection times due to the duration required for analytes to reach surface-bound receptors [42–44]. Harnessing the advantage of MNPs, these were spotted in the beginning of the channel, hence allowing analyte–antibody interactions throughout the flow through the channel. This was compared to MPNs spotted in the detection zone to demonstrate the advantage of prolonged incubation and diffusional access between capture antibody and analyte (ESM, Fig. S4). The concentration of capture Abs, Ab-fluorescent NPs, and MNPs strongly influenced the sensitivity of the immunoassay. For a 15 µl NT-proBNP-spiked HEPES buffer (1 ng∙ml^−1^), we found optimal concentrations of 2.5 µg∙ml^−1^, 2% (w/v) and 3 µg, respectively (Fig. 3a–c). It is worth noting that as the concentration of MNPs increases, the fluorescence intensity also increases. This is due to the increased density of streptavidin recognition sites for the specific binding of the biotinylated sandwich complex, which leads to an improvement in the detection sensitivity. To further optimize the diffusional transport to the reactive sites, the duration for which the MNPs are in contact with the sample was modified by changing the flow rate as this influences capture efficiency, analyte–antibody interaction, and shear forces [45]. Thus, the fluorescence signal after the immunoreaction for a given analyte concentration at different flow rates was investigated (Fig. 3d). First, 15 µl of NT-proBNP-spiked HEPES buffer (1 ng∙ml^−1^) was applied on the ready-to-go lateral flow channel, which subsequently initiated the rehydration of the dried reagents. The sample was transported with flow rates of 4, 3, 2, and 1 µL∙min^−1^, and the fluorescence intensity was measured at the detection zone. It was found that lower flow rates resulted in overall more favorable conditions combining superior capture efficiency and increased interaction time. Furthermore, the rehydration ability of the dried reagents such as capture Abs, Ab-fluorescent NPs, and MNPs was visually observed to be better at lower flow rates due to the longer rehydration times. In general, the highest fluorescence signal was obtained with the optimal flow rate of 2 µl∙min^−1^.

### Performance of the lateral flow channel assay

**Fig. 4:**
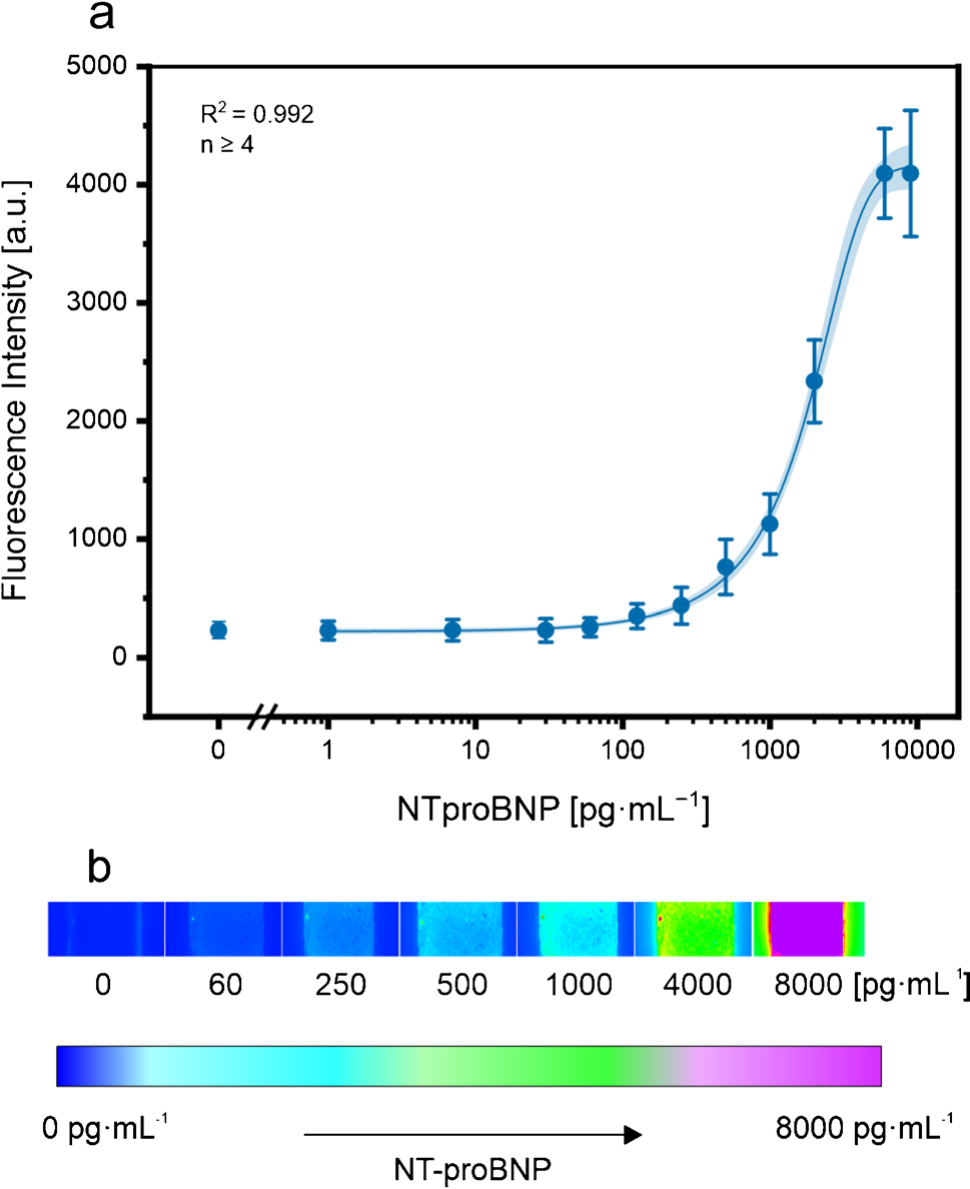
Dose–response curve of NT-proBNP concentration in undiluted whole blood with logistic fit (blue line), 95% confidence interval (shaded blue curve) (**a**), and fluorescence images representing NT-proBNP concentration (**b**). Standard deviations were calculated based on three parallel measurements using four flow channels, while outliers were removed after *Q*-test (confidence interval 95%). Error bars represent mean values ± 1 σ (*n* = 3)

Finally, the previously optimized conditions were transferred to generate dose–response curves for NT-proBNP in buffer and undiluted human blood. The spiked buffer measurements yielded a LOD of 26.7 pg∙mL^−1^ with an error of only 11% (*n* = 4) (data not shown). In spiked human blood, a LOD of 43.1 pg∙mL^−1^ and a mean standard deviation of 18% (*n* = 4) were obtained (Fig. 4). As anticipated, the sensitivity and standard deviation were slightly affected due to the partial blocking of MNPs by adsorbed serum proteins and an increased background signal originating from these proteins. Notably, the mean error of the proposed assay is expected to benefit from enhanced fabrication processes when specifically tailored for large-scale production. In contrast to the established POCT assays presently employed in clinical environments, like the commercially accessible NT-proBNP tests developed by Roche Diagnostics [46], one of the key benefits of our assay is its ability to deliver similar results (detection range, 60–9000 pg∙mL^−1^) using a minimal sample volume of just 15 µL. This is a remarkable improvement compared to the Roche Cobas h 232 that requires up to 10 times more sample volume (15 µL vs. 150 µL). While experiments revealed that the limit of detection decreases with higher volume input, the primary objective was to showcase the viability of using small volumes. Specifically, our sensor can detect 0.076 fmol of NT-proBNP, whereas the Roche Cobas h 232 detects 1.06 fmol. To facilitate accurate addition of 15 µL by the patient, the implementation of capillary tubes, a conventional method for precise measurement of sample volumes, is also envisioned for the diagnostic test. This advancement not only preserves high sample volumes but also simplifies the sampling process, making it more convenient for both medical assistants and patients. Additionally, the utilization of a bead-based immunoassay in this lateral flow channel overcomes the limitations associated with diffusion in traditional microfluidic sensors. By leveraging the unique properties of magnetic nanoparticles, the assay enables efficient and targeted analyte capture and enhancing sensitivity. Recently, a newly commercially available MNP-based NT-proBNP assay has been demonstrated that is technologically the closest to our lateral flow channel assay [47]. This microfluidic chip enables NT-proBNP detection from 20 µL blood within 12 min with a range of 50–9000 pg∙mL^−1^. The assay demonstrated an average error of 4.1% within the NT-proBNP range of 59–4559 pg∙mL^−1^, indicating its superior analytical precision. However, the testing protocol relies on additional washing steps, which in turn raises the complexity and expenses associated with the assay. Another similar MNP-based system is the well-established Cobas Elecsys® for the ultra-sensitive detection of NT-proBNP [48]. The platform provides a superior detection range of 5–35,000 pg∙mL^−1^ with only 15 µL sample and an assay time of 9–18 min. Nevertheless, this system operates within a laboratory setting, being costly, necessitating sample preparation, and unsuitable for point-of-care implementation. The materials utilized in LFAs, such as fleece fiber and membrane materials, often exhibit variations and inconsistencies in fabrication process. These variations can impact factors such as sample flow rate and the release of dried reagents, ultimately affecting sensor performance. Thus, the incorporation of a compact pump system for controlled and automated fluid transport in this assay provides a crucial advantage in terms of precise sample transportation. Despite the need for an additional handheld device, this controlled flow system ensures consistent and uniform sample movement, reducing the impact of sample variability and improving the reliability of results. This feature is particularly important in applications where accurate and reproducible measurements are paramount.

### Signal stability

**Fig. 5:**
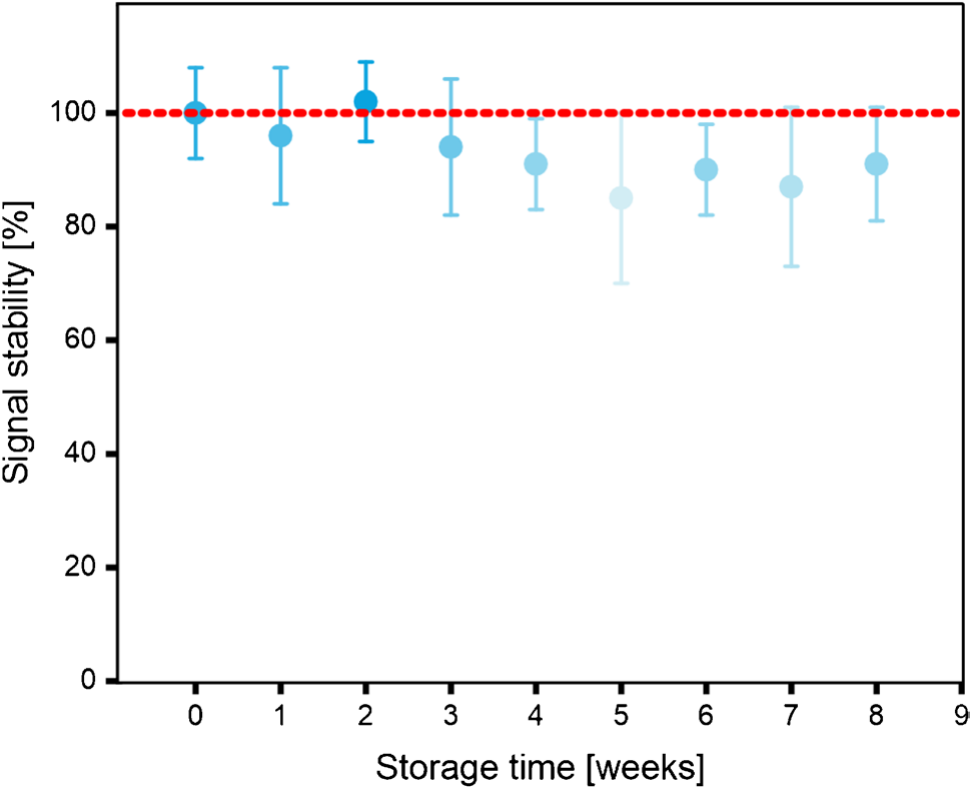
Performance stability of the bioassay with dried capture Abs, Ab-fluorescent NPs in 6% (w/v) sucrose, and MNPs in 6% (w/v) dextran at 50 °C, stored at 4 °C over 8 weeks. Standard deviations were calculated based on three parallel measurements on three different LFAs, while outliers were removed after *Q*-test (confidence interval 95%). Error bars represent mean values ± 1 σ (*n* = 3). In all measurements, the immunoassay was performed in the lateral flow channel with a constant analyte concentration of 1 ng∙mL^−^1, and signal stability means were normalized to the signal right after drying (storage time = 0)

Furthermore, the signal stability of the one-step NT-proBNP immunoassay was assessed over a period of 8 weeks, using all the required reagents stored in a dry state. It is well-established that storing the reagents in the conjugate pad within a storage buffer, composed of stabilizers like trehalose or sucrose, preserves the biological activity of antibodies and ensures colloidal stability [49, 50]. In our assay, the reagents were dried on the sensor support, in direct contact to the PET surface. Within this hydrophobic environment, proteins might experience unfavorable conformational changes or irreversible adsorption, leading to compromised protein functionality and, consequently, hindering the overall sensor performance [51–53]. Moreover, it was crucial to enable fast rehydration and maintain the colloidal stability of the fluorescence–polystyrene nanoparticles and the MNPs. Particularly, MNPs have a tendency to create permanent aggregates when there is a transition in the medium composition, for instance, from liquid to dry [54]. Thus, the study was essential for evaluating the storage stability of the utilized reagents after drying on the sensor surface. Therefore, the capture Abs and the fluorescence-NPs were dried in 6% (w/v) sucrose, and the MNPs were dried in 6% (w/v) dextran at 50 °C for 10 min for the long-term storage. The LFA was stored airtight at 4 °C, and an immunoassay was performed weekly throughout the duration of the study (Fig. 5). During the observation period, the fluorescence signal remains consistent with a mean relative error of 12%. This finding holds significant importance as it validates the overall viability of storing the reagents in a dry state in ambient conditions. In addition to the small number of replicates, the manual fabrication inaccuracies of the lateral flow channel assay have contributed to the overall high error bars.

## Conclusion

In the future of medical care, an increasing need for reliable tests performed without medical personnel yet providing the quantitative and highly sensitive characteristics of standard laboratory tests is predicted not necessarily limited to rural areas and homecare. To fully support this need with POCTs, it is essential to switch from a venous blood draw to a small finger-prick blood sample. This change reduces invasiveness, ensures patient comfort, and streamlines sample handling. Thus, future tests must be as easy-to-use as the one-step LFA yet provide better analytical figures of merit, considering patient comfort. The test system proposed here suggests that through small associated hardware such analytical features are achievable. The combination of nanotechnology and active fluid control along with fluorescence detection has created new possibilities for achieving highly sensitive and reliable point-of-care diagnostics, even if instrument-free visual detection is here no longer possible. Yet, improved surface-to-volume ratios, enhanced reaction kinetics, precise fluid control, and amplified detection have collectively enabled a user-friendly, one-step automated POCT system. Future improvements encompass expanding the scope of detectable analytes, integrating multiplexing capabilities, and developing a cost-effective handheld and portable analytical device that combines fluorescence detection, flow control, and automated analysis. This will allow for easy and efficient testing for both clinical and home use.

### Supplementary Information

Below is the link to the electronic supplementary material.

Supplementary file1 (DOCX 242 KB)
