# Supplementary material for: NT-proBNP detection with a one-step magnetic lateral flow channel assay
Source: Anal Bioanal Chem. 2024 Mar 8;416(10):2411–22. doi: 10.1007/s00216-024-05223-x (PMC10973066; doi:10.1007/s00216-024-05223-x)
Supplement: Supplementary file 1 — Supplementary file1 (DOCX 242 KB) [file 216_2024_5223_MOESM1_ESM.docx]

Supporting Information for

NT-proBNP Detection with a One-Step Magnetic Lateral Flow Channel Assay

Dan Strohmaier-Nguyen ^b^, Carina Horn ^b^, Antje J. Baeumner ^a^^[[1]](#footnote-1)^

*^a^ University of Regensburg, Institute of Analytical Chemistry, Chemo- and Biosensors, 93043 Regensburg*

*^b^ Roche Diagnostics, 68305 Mannheim, Germany*

Table of content

- Text S1. Material and Methods
- Figure S1 – S4
- References

**Text S1. Material and Methods**

**Fluid control system**

The fluid control consisted of a metal housing that includes 1) a LFA mold, 2) an Arduino Due board, 3) and a miniaturized vacuum pump. The fluid control system was linked to a computer via USB and managed using an Arduino code. To initiate the immunoassay the fluid control was mounted on top of the outlet port of the LFA. The piezo effect- based micropump, mechanically positioned on the test strip using a suction cup. Specifically, two piezoceramics in a pump deform when a voltage is applied, allowing the transportation of liquid into a specific direction. Similar principles can be found commercially and described in literature [1].

**Supporting figures**

| 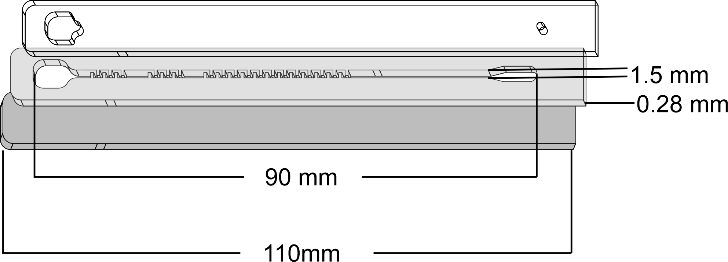 |
| --- |

**Fig. S1** Schematic representation of the three-layered lateral flow channel with Melinex® 329 foil 175 µm as the support layer (dark grey), Melinex®329 foil 250 µm with double-sided adhesive tape as spacer foil (light grey) and Hostaphan RN 100, 125 µm as the cover foil (transparent)

| 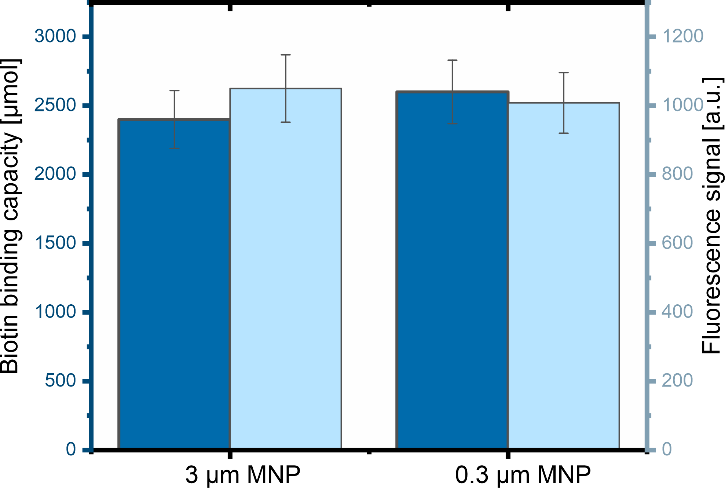 |
| --- |

**Fig. S2** Biotin-binding capacity of large MNPs (3 µm, 2% (w/v)) and small MNPs (0.3 µm, 2 % (w/v)) (dark blue) and assay signal for constant MNP concentration (2 % (w/v)) for a given analyte concentration (c(analyte)=1 ng∙mL^-1^) (light blue). Standard deviations were calculated based on three parallel measurements on three different lateral flow channels (n=3)

| 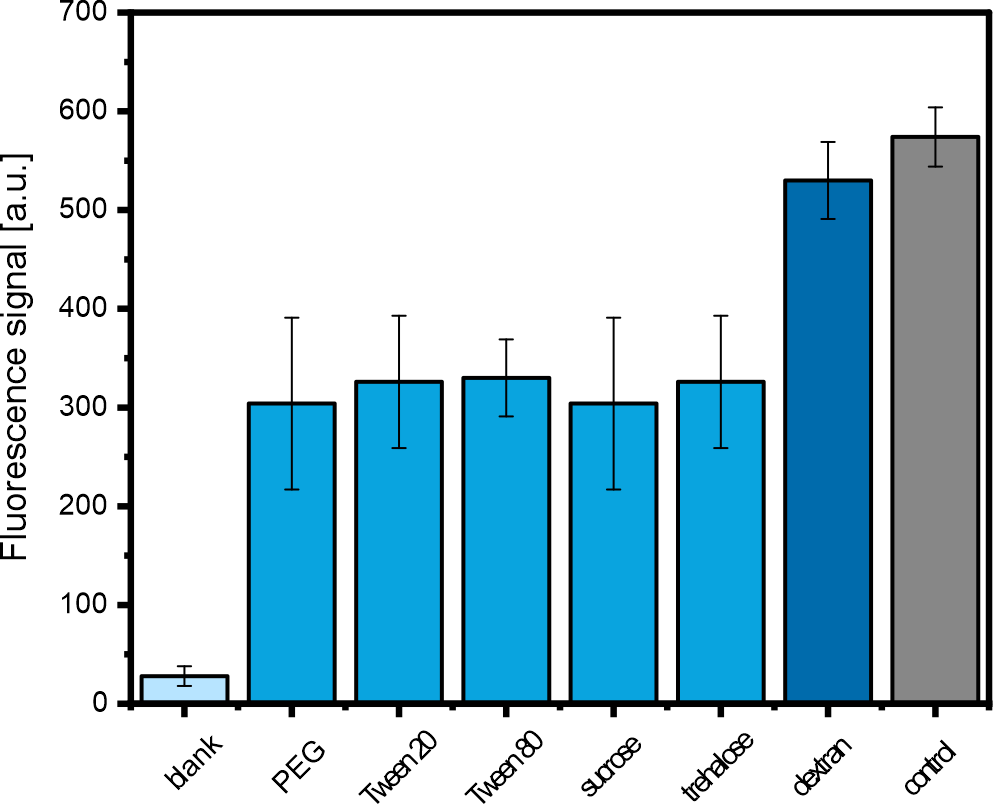 |
| --- |

**Fig. S3** Optimization of drying MNPs with different additives in the dispensing buffer. Plot of the assay signal against the additives. Blank represents dried MNPs in HEPES buffer only, control represents MNPs not dried. 2 µL of MNPs (2 % (w/v) were dried in HEPES dispensing buffer on the sensor support at 50 °C for 10 min with the additives PEG 8000 (5 % (w/v)), Tween 20 (2 % (w/v), Tween 80 (2 % (w/v)), sucrose (20 % (w/v), trehalose (20 % (w/v), dextran (6 % (w/v), respectively. Rehydration was performed with 15 µL BNP-spiked HEPES buffer (1 ng∙mL^-1^) with cAb (2.5 µg∙mL^-1^) and dAb-FNPs (2 % (w/v)) for 5 min. Standard deviations were calculated based on three parallel measurements on three different lateral flow channels (n=3)

| 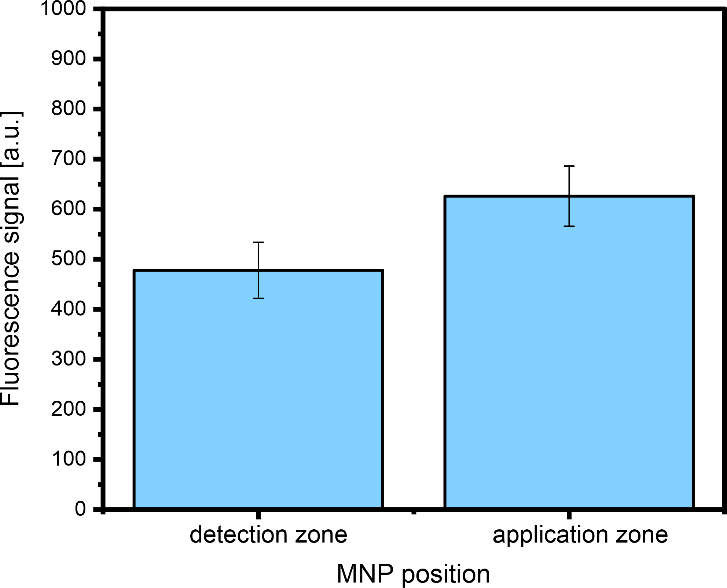 |
| --- |

**Fig. S4** Influence of the deposition area of MNPs (2 % (w/v)) on the immunoreaction (c_[analyte]_ = 1 ng∙mL^-1^, time = 10 min). MNPs deposited and dried in the detection zone have short immunoreaction time and low capture efficiency, resulting in low signal intensities. MNPs deposited upstream next to the sample application area have longer immunoreaction time and higher capture efficiency, resulting in higher signal intensities. Standard deviations were calculated based on three parallel measurements on three different lateral flow channels (n=3)

**References**

1. Li H, Liu J, Li K, Liu Y (2021) A review of recent studies on piezoelectric pumps and their applications. Mech Syst Signal Process 151:107393. https://doi.org/10.1016/j.ymssp.2020.107393

1. Corresponding author.

   E-mail address: [antje.baeumner@ur.de](mailto:antje.baeumner@ur.de) (A.J. Baeumner) [↑](#footnote-ref-1)
